# Study on the Accuracy of Miniprobe Ultrasonic Gastroscopy in Determining the Depth of Infiltration of Early Gastric Cancer

**DOI:** 10.1155/grp/6627449

**Published:** 2025-11-19

**Authors:** Xuan Bai, Yu Zhang, Jingzhai Wang, Pengli Zhang, Tian He, Kelin Yue, Qiang Guo, Zan Zuo

**Affiliations:** ^1^Department of Gastroenterology, The First People's Hospital of Yunnan Province, Kunming, Yunnan, China; ^2^Yunnan Digestive System Disease Clinical Medical Center, Yunnan Digestive Disease Quality Control Center, Kunming, China; ^3^Faculty of Life Science and Technology, Kunming University of Science and Technology, Kunming, China; ^4^Medical School, Kunming University of Science and Technology, Kunming, China

**Keywords:** accuracy, depth of infiltration, early gastric cancer, miniprobe ultrasonic gastroscopy

## Abstract

**Objectives:**

T staging is required before choosing the treatment modality for early gastric cancer (EGC). Ultrasound endoscopy (EUS) is a commonly used method for T staging of EGC. However, the results of studies on the accuracy of EUS in diagnosing the depth of EGC infiltration have been inconsistent. The purpose of this article is to investigate the overall accuracy of miniprobe EUS for diagnosing EGC infiltration depth and to explore the risk factors affecting the accuracy of miniprobe EUS.

**Methods:**

We retrospectively analyzed the data of 136 patients with EGC, all of whom underwent preoperative EUS T staging and pathological specimens were obtained by endoscopic submucosal dissection (ESD) or radical surgery. The accuracy of miniprobe EUS in determining EGC was assessed by comparing the concordance between EGC EUS T staging and histopathological diagnosis and analyzing the lesion characterization factors affecting the accuracy of EUS.

**Results:**

By analyzing the 136 EGC cases included, the overall accuracy of miniprobe EUS for EGC T stage was 77.2% (105/136); the accuracy of miniprobe EUS for diagnosing T1a and T1b EGC was 80.7% (92/114) and 59.1% (13/22), respectively. Lesions combined with ulcers (OR: 3.221; 95% CI: 1.084–9.570; *p* = 0.035) and lesions with depressed morphology (OR: 3.869; 95% CI: 1.208–12.389; *p* = 0.023) were independent risk factors for overdetermination of EGC infiltration depth by miniprobe EUS. *Helicobacter pylori* (*HP*) presenting infection was significantly associated with EGC combined with ulcers (OR: 19.725; 95% CI: 2.451–158.747; *p* = 0.005).

**Conclusions:**

The accuracy of miniprobe EUS in diagnosing T1a EGC is high, and the accuracy in diagnosing T1b EGC is relatively low. Miniprobe EUS is less accurate in determining the depth of infiltration of EGCs combined with ulcers and with a depressed morphology. EGCs are more prone to be combined with ulcers in the setting of a presenting infection of *HP*, which led us to propose the hypothesis “is it possible to improve the accuracy of EUS by short-term eradication of *HP* to reduce the inflammation of EGC ulcers.”

## 1. Introduction

Gastric cancer (GC) ranks fifth for incidence and fourth for mortality with over 1 million new cases and 768,000 deaths worldwide in 2020. The diagnosis of early lesions is essential in order to offer a definitive mini-invasive treatment and improve survival (1). Currently, endoscopic mini-invasive treatment for early gastric cancer (EGC) mainly includes endoscopic mucosal resection (EMR) and endoscopic submucosal dissection (ESD). ESD has been widely used and has become the preferred treatment for EGC within the indications. In order to determine whether EGC is eligible for endoscopic treatment, it is also crucial to clarify the depth of infiltration of the lesion, in addition to evaluating the histopathologic type and size of the lesion and whether it is combined with ulcers (2).

Image enhanced endoscopic technology improves detection of gastric intestinal metaplasia, dysplasia, and EGC (3). Endosonography (EUS) also is nowadays largely available, and its use has been proposed to assess the depth of the invasion of EGC. However, the accuracy of EUS in determining the depth of EGC is currently controversial. Some studies have shown that diagnostic accuracy of overall T staging for EUS varied between 65.0% and 92.1% (4).

The aim of this study is to investigate the application value of miniprobe EUS in the diagnosis of infiltration depth of EGC and to evaluate the risk factors potentially affecting the accuracy of infiltration depth of EGC diagnosed by miniprobe EUS, in order to guide the avoidance of the potential risk factors in the clinical work, and to improve the diagnosis and treatment effect of EGC.

## 2. Materials and Methods

### 2.1. Case Collection and Study Designs

We collected 136 cases of EGC (defined as cancer tissue confined to the gastric mucosal layer or submucosal layer, with or without lymph node metastasis) who underwent ESD or radical surgical treatment at our hospital from July 2019 to July 2024 and were diagnosed with EGC (defined as cancer tissue confined to the gastric mucosal layer or submucosal layer, with or without lymph node metastasis) by pathology. One hundred and thirty-six patients underwent preoperative miniprobe EUS examination, and we retrospectively analyzed the clinical data of these 136 patients with EGC and compared the miniprobe EUS staging with the final histopathologic staging to clarify the risk factors affecting the accuracy of miniprobe EUS in determining the depth of EGC infiltration. Of the 136 lesions in this study, 100 underwent ESD and 42 were treated with radical surgery (six of which were additional surgical procedures after ESD). The study was approved by the Institutional Review Board and complied with the ethical standards of the Declaration of Helsinki.

### 2.2. Equipment and Endoscopist

All 136 EGCs were visualized by white light endoscopy (Device a: H260 or H290, Olympus Medical Systems, Japan; Device b: EG-580N or EG-760, FUJIFILM Corporation, Japan), followed by miniprobe EUS (Device a: UM-2R, 20 MHz, Olympus, Japan; Device b: SP702, 20 MHz, FUJI, Japan). The miniprobe EUS examination was performed by three endoscopists (A, B, and C) with more than 3 years of experience in EUS operation.

### 2.3. Definitions

#### 2.3.1. Grouping of the Lesions

Patients were categorized into *Helicobacter pylori* (*HP*) current infection group and *HP* noncurrent infection group (including *HP* negative and *HP* previous infection) according to the C14 breath test at the time of miniprobe EUS examination. The lesions were broadly typed according to the Paris typing, elevated (0-I), flat (0-II), and depressed (0-III), and in the experiment, 0-I and 0-II in the Paris typing were collectively referred to as nondepressed and categorized as the nondepressed group, while 0-III was categorized as the depressed group. According to the location of the stomach where the lesion was located, it was categorized into four groups: gastric antrum, gastric angle, gastric body, and cardia/fundus. EGC resection specimens with a thickness of 2 mm were stained with hematoxylin and eosin and evaluated pathologically by an experienced gastrointestinal pathologist. Highly or moderately differentiated tubular adenocarcinomas and papillary adenocarcinomas were classified as differentiated, and lowly differentiated tubular adenocarcinomas and imprinted cell carcinomas were classified as undifferentiated. The EGC lesions combined with ulcers were characterized as either surface-adherent thick white mosses in the active stage or mucosal tangles in the healing stage.

#### 2.3.2. Determination of the Depth of Lesion Infiltration

The depth of invasion was assessed according to the typical five-layer structure of the gastric wall presented by EUS, with hypoechoic disruption of Layers 1 and 2 of the gastric wall on the image for invasion of the mucosal layer (EUS-M), disruption of Layer 3 for invasion of the submucosal layer (EUS-SM), disruption of Layer 4 for invasion of the lamina propria (EUS-MP), and disruption of Layer 5 for invasion of the plasma layer (EUS-SS). According to the T staging of GC, the M layer corresponds to T1a and the SM layer to T1b.

### 2.4. Statistical Methods

Correlations between the two variables were tested using the chi-square test or Fisher's exact test. Multivariate tests using multivariate logistic regression analysis were performed to identify risk factors that have an impact on the accuracy of miniprobe EUS in determining EGC infiltration depth. SPSS Package Version 21.0 (SPSS, Chicago, Illinois, United States) was used for statistical analysis.

## 3. Results

### 3.1. Basic Characteristics of Cases

One hundred and thirty-six patients with EGC were analyzed. The mean age of the patients was 60.6 years (range: 35–78 years), and 92 (66.4%) were male. Eighty-three cases (61.0%) were *HP*-positive, the mean diameter of the tumors was 2.1 cm (range: 0.4–4.6 cm), and the tumors were combined with ulcers in 25 (18.4,%); the histopathological diagnosis was differentiated in 124 cases (91.2%) and undifferentiated in 12 cases (8.8%). One hundred fourteen lesions were histopathologically diagnosed as having an infiltration depth of M (83.8%), and 22 lesions had an infiltration depth of SM (16.2%). One hundred (69.1%) of the patients initially underwent endoscopic resection, with six patients with submucosal infiltration undergoing additional surgical treatment (Table 1).

### 3.2. Diagnostic Accuracy of Miniprobe EUS for T Staging of EGC

The overall accuracy of miniprobe EUS for EGC T staging was 77.2% (105/136). The accuracy of EUS in diagnosing T1a and T1b EGC was 80.7% (92/114) and 59.1% (13/22), respectively. Of the 136 EGC cases, histopathologic diagnosis of mucosal layer carcinoma was made in 114 cases, and 92 mucosal layer lesions (80.7%) were accurately diagnosed as EUS-M by miniprobe and 22 were overestimated as EUS-SM. Histopathologic diagnosis of submucosal carcinoma was made in 22 cases; miniprobe EUS accurately diagnosed 13 lesions (59.1%); six lesions (27.3%) were underestimated as EUS-M, and three lesions (13.6%) were overestimated as EUS-MP. The sensitivity, specificity, positive predictive value (PPV), and negative predictive value (NPV) of miniprobe EUS for the diagnosis of EGC in the mucosal layer were 80.7%, 68.4%, 93.9%, and 37.1%, respectively (Tables 2 and 3).

### 3.3. Analysis of Risk Factors Affecting the Accuracy of Miniprobe EUS in Determining the Depth of EGC

In univariate analysis, the presence of *HP* presenting infection in the background of gastric mucosa, lesion diameter > 2 cm, lesion combined with ulcer, and lesion with depressed morphology had an effect on the accuracy of miniprobe EUS in diagnosing the depth of EGC infiltration. Subsequently, multivariate logistic regression analysis was performed, and the accuracy of miniprobe EUS in diagnosing EGC was significantly correlated with lesions combined with ulcers (odds ratio [OR]: 3.221; 95% CI: 1.084–9.570; *p* = 0.035), and lesions with a depressed pattern (OR: 3.869; 95% CI: 1.208–12.389; *p* = 0.023) were significantly associated. However, *HP* infection status, tumor size, location, and histopathological type did not significantly affect the accuracy of miniprobe EUS. Other factors such as ultrasound endoscopist and ultrasound endoscopy equipment were not associated with the accuracy of EUS (Tables 4 and 5).

### 3.4. Analysis of Risk Factors for Overestimation and Underestimation of Infiltration Depth in EGC by Miniprobe EUS

In univariate analysis, miniprobe EUS tended to overestimate EGC T staging for EGCs with *HP* presenting infection in the gastric mucosal background, lesions combined with ulcers, and lesions with a depressed morphology and underestimated EGCs with a diameter of > 2 cm. Subsequently, multivariate logistic regression analysis was performed, and miniprobe EUS tended to overestimate EGC T staging for lesions combined with ulcers (OR: 3.483; 95% CI: 1.145–10.591; *p* = 0.028) and lesions with a depressed morphology (OR: 3.839; 95% CI: 1.092–13.500; *p* = 0.036). The underestimation of EGC T staging by miniprobe EUS was independent of the macroscopic type of the EGC, its size, the presence or absence of a combined ulcer, the type of histopathology, and the presenting *HP* infection (Tables 6 and 7).

### 3.5. Relationship Between *HP* Infection and Characteristics of EGC Lesions

Multifactorial logistic regression analysis showed that *HP* presenting infection was significantly associated with EGC combined with ulceration (OR: 19.725; 95% CI: 2.451–158.747; *p* = 0.005) and with histopathologically differentiated EGC (OR: 5.693; 95% CI: 1.284–25.232; *p* = 0.022), and *HP* presenting infection was not associated with tumor size and depressed morphology (Tables 8).

## 4. Discussion

EUS has been widely used for T staging of gastrointestinal tumors, but the results of the current studies on the accuracy of EUS for T staging of gastrointestinal tumors vary. Some studies have suggested that there is little difference between the role of CT and EUS in T staging, and the overall accuracy of spiral CT and EUS for T staging was 69.4% and 70.4% (5). Differently, it has also been suggested that EUS is considered to be the most accurate method of localized regional staging and is commonly used to differentiate between mucosal and submucosal lesions. In contrast, computed tomography (CT) is used to detect the presence of distant metastases (6). Some studies have concluded that endoscopic ultrasonography is less accurate in staging the depth of invasion of EGCs and that EUS may not be a necessary routine test before endoscopic resection of EGC (7, 8). However, it has also been suggested that endoscopic ultrasonography is a useful modality in accurately determining the invasion depth of EGC before ESD (9). The accuracy of miniprobe EUS was significantly higher than that of radial EUS (*p* < 0.001) (8). In our study, 20-Hz miniprobe EUS was used, and the results showed that the overall accuracy of miniprobe EUS in diagnosing the infiltration depth of EGC was 77.2% (105/136), and the accuracy of miniprobe EUS in diagnosing T1a and T1b EGC was 80.7% (92/114) and 59.1% (13/22), respectively. It can be seen that miniprobe EUS has a high accuracy in diagnosing EGC with infiltration depth of T1a, while the accuracy for T1b is relatively low. Therefore, miniprobe EUS should be more cautious in diagnosing EGC with T1b.

In terms of risk factor studies for the accuracy of EUS in diagnosing the depth of EGC infiltration, it has been suggested that tumor size ≥ 2 cm, the presence of ulceration, nonflat morphology, and undifferentiated histological type are significantly associated with the accuracy of endoscopic ultrasonography (8–11). Our study further supplements these findings by demonstrating, through multivariate logistic regression analysis, that lesions combined with ulcers and lesions with a depressed morphology are independent risk factors for reducing the accuracy of miniprobe EUS. Specifically, these two factors increase the risk of overestimation by 3.2 times and 3.8 times, respectively. This quantitative analysis provides a more precise reference for clinical decision-making when evaluating such lesions. For example, in clinical practice, for EGCs with these characteristics, combining with magnifying endoscopy or targeted biopsy may help improve the diagnostic accuracy. In EGCs with lesions ≥ 2 cm in extent, the effective scanning range of miniprobe EUS is relatively smaller as the lesion length increases, potentially missing localized submucosal infiltrating lesions; however, it has been shown that there is no relationship between lesion size and the accuracy of EUS (11). A large-sample study conducted by Choi et al. showed that the staging accuracy of EUS in differentiated cancers was 70.9%, whereas in undifferentiated cancers the staging accuracy could be reduced to 59.1% (8). This may be due to the difference in biological behavior between differentiated and undifferentiated carcinomas; undifferentiated carcinomas tend to histologically show infiltration of single or nested tumor cells, a feature that is difficult to detect by EUS (12). However, another study again showed no relationship between EGC histopathologic type and the accuracy of EUS (10, 11). Tsuzuki et al. concluded that the diagnostic accuracy of the invasion depth diminished for lesions in the upper third of the stomach (13). The relatively thin submucosal layer of the upper third of the stomach with its abundant vascular system would affect the staging accuracy of EUS (14, 15). However, some studies have shown no relationship between the location of the lesion and the accuracy of EUS (8, 16, 17). Our study showed that the accuracy of miniprobe EUS in diagnosing the depth of EGC infiltration was significantly correlated with lesions combined with ulcers (*p* = 0.035) and lesions with a depressed morphology (*p* = 0.023). In both cases, the accuracy of miniprobe EUS in determining the depth of EGC infiltration was poor, which may be related to the significant infiltration of inflammation or fibrosis and adhesion at the base of the lesion, and it was more difficult for EUS to distinguish whether it was a tumor infiltration or an inflammatory infiltration.

Neutrophil infiltration was mild in the *HP* eradication group (18). *HP* eradication has been reported to flatten lesions of differentiated-type EGC (19, 20). In this study, *HP* presenting infection was also included as an independent variable, in order to clarify whether the lesion would affect the EUS judgment of EGC infiltration depth due to inflammatory infiltration caused by *HP* infection. In the univariate study, we found that the presence of *HP* current infection in the background of the gastric mucosa had an effect on the accuracy of EUS in diagnosing the depth of EGC infiltration (*p* = 0.033), while in the multifactorial analysis, *HP* presenting infection had no effect on the accuracy of EUS diagnosis of EGC infiltration depth (*p* = 0.264). This situation may be due to the covariance of *HP* presenting infection with the other independent variables and the correlation with the other independent variables. Therefore, we explored the correlation between *HP* presenting infection and other independent variables and found that *HP* presenting infection was significantly correlated with EGC combined with ulcers (*p* = 0.005), which suggests that *HP* infection may indirectly affect the accuracy of EUS by promoting ulcer formation. Given that existing studies have confirmed that gastric mucosal inflammation can subside significantly within 4 weeks after *HP* eradication (21, 22), we propose a hypothesis to be verified: short-term *HP* eradication may improve the accuracy of EUS in judging the infiltration depth by reducing inflammation in ulcerated areas. However, this hypothesis needs to be directly confirmed by future prospective studies, such as comparing EUS findings before and after eradication therapy in *HP*-positive EGC patients with ulcers. According to a previous study, many of the *HP*-negative patients had undifferentiated-type cancer (23), which is consistent with our study.

Several strengths exist in this study. First, we assessed the accuracy of EUS in determining EGC T staging. Second, risk factors for the accuracy of EUS in determining EGC T staging were clarified by multifactorial analysis. Finally, we analyzed the relationship between *HP* and lesions of EGC combined with ulcers and proposed the hypothesis that short-term eradication of *HP* has the potential to reduce the inflammatory potential of EGC ulcers. At the same time, this study has a few limitations. First, the study was a single-center study with a small sample size of EGCs with infiltration depth of SM (*n* = 22), which may have some risk of sample bias. This is consistent with the relatively low incidence of T1b EGC in clinical practice (about 15%–20%) (8, 11). Our study conducted a multivariate analysis of 136 cases of EGC, identifying ulcer-combined lesions and depressed-type lesions as independent risk factors affecting the accuracy of miniprobe EUS. These findings can provide certain references for EUS diagnosis of infiltration depth in EGCs, including T1b lesions. In the future, multicenter studies with larger sample sizes of T1b lesions are needed to further validate our conclusions. Second, the study was a retrospective analysis, and the cases collected only included EGCs that underwent ESD or radical surgical procedures after having preoperative EUS. EGCs without preoperative EUS were not included in the statistics.

In summary, miniprobe EUS has high accuracy in diagnosing EGCs with infiltration depth of T1a and relatively low accuracy in diagnosing EGCs with infiltration depth of T1b. EGCs combined with ulceration and depressed morphology are risk factors affecting EUS in determining their infiltration depth, and EUS may not be the best choice in these two cases, and there is a need to avoid judging the infiltration depth of the lesion too deeply, which may lead to the patient undergoing unnecessary surgery. In EGCs with lesions combined with ulcers, whether short-term *HP* eradication followed by EUS can reduce ulcer inflammation and thus improve the diagnostic accuracy of EUS for this type of lesion remains to be further studied.

## Figures and Tables

**Table 1:** Basic characteristics of patients with early gastric cancer and lesions.

**General patient characteristics (** **n** = 136**)**	**n** **(%)**
Age, median (range) (years)	60.6 (35–78)
Gender (male/female) (%)	92 (67.6%)/44 (32.4%)
Characteristics of early gastric cancer (*n* = 136)	
*HP* infection at ultrasound scanning	*n* (%)
*HP*-positive	83 (61.0%)
*HP*-negative	53 (39.0%)
Location	
Gastric antrum	74 (54.4%)
Gastric angle	19 (14.0%)
Gastric body	30 (22.1%)
Gastric fundus/cardia	13 (9.5%)
Diameter	
≤ 2 cm	95 (69.9%)
> 2 cm	41 (30.1%)
Ulcer	
Ulcer-negative	111 (81.6%)
Ulcer-positive	25 (18.4%)
Macroscopic type	
Nondepressed	117 (86.0%)
Depressed	19 (14.0%)
Histopathologic type	
Undifferentiated	12 (8.8%)
Differentiated	124 (91.2%)
Ultrasound endoscopist	
A	63 (46.3%)
B	48 (35.3%)
C	25 (18.4%)
mEUS device	
a	88 (64.7%)
b	48 (35.3%)
Resection method	
ESD	100 (73.5%)
Additional surgery after ESD/surgery	42 (30.9%)
Pathologic depth	
M	114 (83.8%)
SM	22 (16.2%)

Abbreviations: ESD, endoscopic submucosal dissection; *HP*, *Helicobacter pylori*; M, mucosa; mEUS, miniprobe ultrasound endoscopy; SM, submucosa.

**Table 2:** Overall accuracy of mEUS in determining the depth of infiltration of early gastric cancer.

**Overall accuracy (** **n** = 136**)**	
Accuracy, *n* (%)	Nonaccuracy, *n* (%)
105 (77.2%)	31 (22.8%)

**Table 3:** Diagnostic accuracy of mEUS for T staging of early gastric cancer.

**EUS-probe T staging**	**Pathological staging, ** **n** **(%)**	
	**M**	**SM**
M	92 (80.7%)	6 (27.3%)
SM	22 (19.3%)	13 (59.1%)
MP	0 (0.0%)	3 (13.6%)

Abbreviations: M, mucosa; mEUS, miniprobe ultrasound endoscopy; MP, muscularis propria; SM, submucosa.

**Table 4:** Univariate analysis of risk factors affecting the accuracy of mEUS in determining the depth of early gastric cancer.

**Characteristics of early gastric cancer (** **n** = 136**)**	**Accuracy, ** **n** **(%)**	**Nonaccuracy, ** **n** **(%)**	**p**
*HP* infection at ultrasound scanning			0.033
*HP*-positive	59 (71.1%)	24 (28.9%)	
*HP*-negative	46 (86.8%)	7 (13.2%)	
Location			0.823
Gastric antrum	55 (74.3%)	19 (25.7%)	
Gastric angle	15 (78.9%)	4 (21.1%)	
Gastric body	24 (80.0%)	6 (20.0%)	
Gastric fundus/cardia	11 (84.6%)	2 (15.4%)	
Diameter			0.012
≤ 2 cm	79 (83.2%)	16 (16.8%)	
> 2 cm	26 (63.4%)	15 (36.6)	
Ulcer			< 0.01
Ulcer-negative	97	14	
Ulcer-positive	13	12	
Macroscopic type			< 0.01
Nondepressed	97 (82.9%)	20 (17.1%)	
Depressed	8 (42.1%)	11 (57.9%)	
Histopathologic type			0.362
Undifferentiated	8 (66.7%)	4 (33.3%)	
Differentiated	97 (78.2%)	27 (71.8%)	
Ultrasound endoscopist			0.701
A	47 (74.6%)	16 (25.4%)	
B	39 (81.2%)	9 (18.8%)	
C	19 (76.0%)	6 (24.0%)	
mEUS device			0.406
a	66 (75%)	22 (25%)	
b	39 (81.3%)	9 (18.8%)	

Abbreviations: *HP*, *Helicobacter pylori*; mEUS, miniprobe ultrasound endoscopy.

**Table 5:** Multivariate analysis of risk factors for the accuracy of mEUS in determining the depth of infiltration of early gastric cancer.

	**OR**	**95% CI**	**p**
*HP* infection at ultrasound scanning			
*HP*-positive	1	—	—
*HP*-negative	1.877	0.621–5.674	0.264
Diameter			
≤ 2 cm	1.972	0.744–5.226	0.172
> 2 cm	1	—	—
Ulcer			
Ulcer-negative	3.221	1.084–9.570	0.035
Ulcer-positive	1	—	—
Macroscopic type			
Nondepressed	3.869	1.208–12.389	0.023
Depressed	1	—	—
Histopathologic type			
Undifferentiated	1	—	—
Differentiated	3.076	0.730–12.955	0.126

Abbreviations: 95% CI, 95% confidence interval; *HP*, *Helicobacter pylori*; OR, odds ratio.

**Table 6:** Univariate analysis of risk factors for overestimation and underestimation of infiltration depth in early gastric cancer by mEUS.

	**Overestimation, ** **n** **(%)**	**p**	**Underestimation, ** **n** **(%)**	**p**
*HP* infection at ultrasound scanning		0.015		0.767
*HP*-positive	21/83 (25.3%)		3/83 (3.6%)	
*HP*-negative	4/53 (7.5%)		3/53 (5.7%)	
Diameter		0.06		0.044
≤ 2 cm	14/95 (14.7%)		2/95 (2.1%)	
> 2 cm	11/41 (26.8%)		4/41 (9.8%)	
Ulcer		< 0.001		0.760
Ulcer-negative	14/111 (12.6%)		5/111 (4.5%)	
Ulcer-positive	11/25 (44.0%)		1/25 (4.0%)	
Macroscopic type		< 0.001		0.055
Nondepressed	16/117 (13.7%)		4/117 (3.4%)	
Depressed	9/19 (47.4%)		2/19 (10.5%)	
Histopathologic type		0.949		0.055
Undifferentiated	2/12 (16.7%)		2/12 (16.7%)	
Differentiated	23/124 (18.5%)		4/124 (3.3%)	

Abbreviation: *HP*, *Helicobacter pylori*.

**Table 7:** Multivariate analysis of risk factors for overestimation and underestimation of infiltration depth in early gastric cancer by mEUS.

	**Overestimation, ** **n** **(%)**			**Underestimation, ** **n** **(%)**		
	**OR**	**95% CI**	**p**	**OR**	**95% CI**	**p**
*HP* infection at ultrasound scanning						
*HP*-positive	2.487	0.686–9.012	0.166	1	—	—
*HP*-negative	1	—	—	1.277	0.172–9.507	0.811
Diameter						
≤ 2 cm	1	—	—	1	—	—
> 2 cm	1.582	0.532–4.707	0.41	4.480	0.702–28.573	0.113
Ulcer						
Ulcer-negative	1	—	—	1	—	—
Ulcer-positive	3.483	1.145–10.591	0.028	1.558	0.111–21.925	0.743
Macroscopic type						
Nondepressed	1	—	—	1	—	—
Depressed	3.839	1.092–13.500	0.036	4.722	0.590–37.797	0.144
Histopathologic type						
Undifferentiated	2.025	0.340–12.064	0.439	6.502	0.806–52.451	0.079
Differentiated	1	—	—	1	—	—

Abbreviations: 95% CI, 95% confidence interval; *HP*, *Helicobacter pylori*; OR, odds ratio.

**Table 8:** Relationship between *HP* infection and characteristics of early gastric cancer lesions.

	**OR**	**95% CI**	**p**
Diameter			
≤ 2 cm	1	—	—
> 2 cm	1.372	0.573–3.285	0.478
Ulcer			
Ulcer-negative	1	—	—
Ulcer-positive	19.725	2.451–158.747	0.005
Macroscopic type			
Nondepressed	1	—	—
Depressed	2.614	0.618–11.065	0.192
Histopathologic type			
Undifferentiated	1	—	—
Differentiated	5.693	1.284–25.232	0.022

## Data Availability

The data that support the findings of this study are available from the corresponding authors upon reasonable request.
